# Experiences of infertility among couples in Morocco

**DOI:** 10.3389/frph.2024.1513243

**Published:** 2025-01-07

**Authors:** Amal Benbella, Gitau Mburu, Asmaa Zaidouni, Sanae Elomrani, Abdelhakim Yahyane, Karima Gholbzouri, James Kiarie, Rachid Bezad

**Affiliations:** ^1^ Assisted Reproductive Technology Center, Maternity and Reproductive Health Hospital les Orangers of IbnSina University Hospital, Rabat, Morocco; ^2^ Faculty of Medicine and Pharmacy, University Mohammed V, Rabat, Morocco; ^3^ UNDP-UNFPA-UNICEF-WHO-World Bank Special Programme of Research, Development and Research Training in Human Reproduction (HRP), Department of Sexual and Reproductive Health and Research, World Health Organization, Geneva, Switzerland; ^4^ Department of Biology and Health, University Ibn Toufail, Kenitra, Morocco; ^5^ Population Directorate, Ministry of Health and Social Protection, Rabat, Morocco; ^6^ Reproductive Health and Research Unit, WHO Eastern Mediterranean Regional Office, Cairo, Egypt

**Keywords:** infertility, qualitative, experiences, Morocco, LMICs, fertility care

## Abstract

**Introduction:**

Infertility has significant implications on health. It affects physical, emotional, and social wellbeing. In Morocco, about 12% of couples or live with infertility. In 2013 the first public Assisted Reproductive Technology (ART) Center was established at the Maternity and Reproductive Health Hospital les Orangers in Rabat, Morocco. This qualitative study explores the experiences of infertility among couples who have benefited from diagnosis and treatment at ART center. The study represents a part of the overall evaluation of this first public ART centre in Morrocco.

**Methods:**

Data were collected through in-depth patient interviews. A semi-structured interview guide was used to elicit the perspectives and experiences couples with infertility. Data collection occurred between January and March 2023. Inductive thematic analysis was conducted to explore the experiences of infertility among the couples.

**Results:**

The study showed that couples with infertility were exposed to negative experiences such as (i) biomedical experiences (diagnostic delays, fragmented care, multiplicity of treatments), (ii) social experiences (gendered social pressures, stigma, discrimination, marital challenges, isolation and disrupted social routines), (iii) mental experiences (anger, anxiety, depression, and mood changes) as well as (iv) financial limitations (out of pocket payments and financial indebtedness). Solutions addressing these challenging experiences were suggested by interviewed couples including continued quality and respectful care to enhance biomedical experiences; public education campaigns to educate the public and destigmatize infertility to improve social experience, inclusion of psychosocial services as part of fertility integrated care to enhance psychological experiences, and enhanced financial protection, and service decentralisation to mitigate negative financial pressures.

**Conclusion:**

Couples with infertility are exposed to various challenges in the biomedical, social, psychological, and financial domains. A health system strengthening approach is essential to address those challenges, and multiple strategies are needed to improve the experiences of couples with infertility in Morocco. Given interconnectedness between biomedical, social, psychological, and financial divers of experiences, a holistic approach is required to comprehensively improve the experiences of infertile couples and address all the issues faced by couples during their treatment journey.

## 1 Introduction

Infertility is defined as a disease of the reproductive system characterised by the failure to achieve clinical pregnancy after 12 months or more of regular unprotected sexual intercourse (1). Recent estimates show that infertility is a common global public health problem, affecting one in six people of reproductive age (2). In addition, prevalence of infertility does not differ significantly between high-income and low- and middle-income countries, demonstrating that it affects people everywhere (2).

Infertility has significant biomedical implications, and can affect physical, emotional and social wellbeing (3). For couples who decide to start a family, difficulty conceiving a child can become a source of tension, conflict (4), financial stress (5), and ultimately poor quality of life (6). Indeed, in most countries, infertility has consequences for families and communities, through its effects on stigma, domestic violence, divorce, polygamy and even suicidal thoughts (7–10).While men often contribute to infertility in a couple, women are often blamed (8, 9), and are often disproportionately stigmatized, disinherited or neglected within families and communities (8–10).

The experiences, financial and social consequences of infertility could differ between countries and cultures (9).However, there is consensus that in all settings, the initial diagnosis of infertility has a profound negative impact on both men and women (3). Additional stress can occur during treatment of infertility (6, 11), and contribute to interruption of care and further delay there solution of infertility (12). Indeed, assisted reproductive technologies (ART) are a source of significant psychological consequences (13).Therefore, holistic understanding of the experience of infertility from diagnosis, through to treatment is important.

In Morocco, infertility is estimated to affect12% of couples, according to a 2015 nationwide survey (14). A recent study showed that 39.6% of infertility cases were due to a female factor, 28.2% had a male factor, 17% had both male and female factors and in 15.2% of couples, the cause of infertility was undetermined (15). Despite this burden of infertility, provision of *in vitro* fertilization (IVF) and other medically assisted reproduction services has not historically been a policy priority (16), as is the case in many other low and middle income countries (17). In recent years however, Moroccan national health sector policies have started to address infertility, with a recent commitment to establish a “National plan for ART” (18), that lead to the development of the first public ART center in Morocco in 2013 (19).

While there is ample evidence that infertility is common in Morocco, little is known about the experiences of infertility among infertile couples in the country. Isolated studies indicate that infertile couples suffer from depression, anxiety (20) and stress (21), while another study found that infertile Moroccan couples are in need of information as well as social, spiritual, emotional and financial support during the infertility treatment (22). Apart from these studies, little other documentation exists in relation to experiences of people with infertility in Morocco.

### 1.1 Study aim

This study aims to obtain an in-depth understanding of couple's experiences of their diagnosis, treatment of infertility in Morocco. The study chronicles patient's experiences of initial diagnosis, health seeking and treatment journey. This was undertaken as part of the evaluation of the first public ART centre, in-order to provide a basis for the development of interventions that can improve the experiences, health, and wellbeing of couples with infertility.

## 2 Methods

### 2.1 Study setting

The study was conducted at the ART center of the Maternity and Reproductive Health Hospital les Orangers (HMSRO) of Ibn Sina University Hospital (CHUIS) in Rabat. The ART center is the first public fertility center in Morocco, which serves patients from different regions of the country.

### 2.2 Study design

This paper is a part of a larger mixed methods evaluation study that utilized both qualitative and quantitative approaches to understand the impact of the center. Findings related to the evaluation of the ART center, including its quantitative clinical outcomes is documented elsewhere (19).In this paper, we report qualitative findings related to the experiences of couples with infertility who have sought services at the centre.

### 2.3 Participant recruitment procedures

A total of 39 participants took part in this study. These participants were recruited from three different population groups to ensure representation and diversity of perspectives, as shown in Table 1.

**Table 1:** Participants in the overall mixed methods study.

Participant type	Sample sizes
Patients receiving fertility care	• 6 couples who have completed treatment (*n* = 12) • 4 couples who are in the process of receiving IVF (*n* = 8) • 4 couples who are on the waiting list for IVF (*n* = 8)
Providers of fertility care	• Medical doctor (*n* = 2) • Midwife (*n* = 1) • Laboratory technician (*n* = 1) • Healthcare provider from referring hospital (*n* = 2)
Policy stakeholders involved in fertility care	• ART Center Director (*n* = 1) • Ministry of Health (*n* = 2) • Public Health expert (*n* = 1) • Professional Fertility Association (*n* = 1)

The three groups of participants were approached through several ways. Researchers approached patients during their routine appointments at the ART Centre and told them about the study and its purposes. Health care providers were approached at the ART Centre during their duties, while policy makers were approached in their offices and informed about the study. Participants therefore constituted a convenient sample, recruited consecutively based on their routine clinic appointments, which is a common recruitment strategy (23). Those who showed interest were provided with a future appointment to meet two researchers, at an agreed time and place where privacy and confidentiality could be ensured, for interviews. Table 2 provides detailed characteristics of the participating couples who were interviewed for their experiences documented in this paper.

**Table 2:** Characteristics of the couples with infertility included in the study.

Couples	Women(*N* = 14)	Men(*N* = 14)
Mean Age (years)	34.5	41.21
Region of Residence		
Tanger-Tetouan-AlHoceima	1	
Fez-Meknes	1	
Rabat-Sale-Kenitra	8	
Beni Mellal-Khenifra	1	
Settat-Casablanca	3	
Educational level		
Illiterate	2	0
Primary	1	2
Secondary	8	8
University level	3	4
Mean duration of infertility (Years)	5	
Profession		
Housewife	11	0
Civil servant	2	6
Freelance profession	1	8
Health insurance		
No Health insurance	4	4
Public insurance	9	9
Private insurance	1	1

### 2.4 Data collection procedures

During the appointment, the researchers went over the purpose of the study and checked that the participants understood the purposes of the study before providing written consent. Consent to record the interview on an audio device and to take notes by hand was sought. A semi-structured interview guide was used to elicit the perspectives and experiences of each participant group (See the interview guide attached as Supplementary Material, Additional file 2 Additional file 3, and Additional file 4). The in-depth interviews dwelt on experiences related to receiving, providing, or developing fertility care at the ART Centre for all groups, as well as experiences with an infertility diagnosis and help seeking more generally, for couples with infertility.

Sampling of participants continued consecutively until the sample size of participants was achieved, based on saturation, or stated size whichever was earlier. As is the practice in qualitative studies (23), saturation was reached once interviews started yielding data which was very similar to the already collected information, and no more new patterns or themes were produced. Interviews lasted between 45 and 60 min and were conducted in either Arabic or French language depending on participants' preferences. The quotes included in the paper are translations in English of the direct quotes from participants. However, some words are in Arabic where translation would not be accurate, or ideal. The translator is proficient in both the target language (Arabic and French) and the analysis language (English). A conceptual translation was made to preserve the integrity of the participants' experiences, where verbatim translation would not have been true to the intended meaning. Data collection occurred between January and March 2023.

### 2.5 Data analysis

The audio files from the interviews were transcribed in full. Inductive thematic analysis was conducted to explore the experiences of infertility among the couples. Researchers read through interview transcripts, making initial notes about the content without any preconceived theories. Pieces of text were coded looking for patterns across the codes and identifying broader themes inductively. Codes and subthemes were developed iteratively while remaining open to discovery as recommended for inductive analysis of qualitative research (23). Similar subthemes were then classified to build up emerging themes, which were continually refined. Themes were clearly defined, and a concise name for each was provided. Two researchers coded independently the set of interview transcripts than compared their coding looking for differences, refining the codes and defining the themes. The two coders agreed on all codes. Illustrative quotes were selected for display to support the identified themes. Reporting follows consolidated criteria for reporting qualitative studies (COREQ) (24).

### 2.6 Data presentation

Quotes are presented to illustrate the themes, and the person speaking is identified as either the wife (W) or husband (H) in a couple. Couples were denoted with a “C” and numbered 1–14 based on de-identified codes. [Code = C (Cn; *n* = 1 to *n* = 14)].

### 2.7 Ethical considerations, approval

The study was conducted in accordance with the Declaration of Helsinki and adhered to the principles of research involving human participants. Informed consent was obtained from each participant. Prior to consenting, participants were given full explanations on the purpose of the study and were reassured that their personal details would remain confidential. Researchers informed the patients that their participation was entirely voluntary and reassured them that they were not obliged to participate in this study in-order to obtain services at the Centre. Participants were informed that should they chose not to participate, there would be no consequences whatsoever, and that they retained the right to refuse to answer any questions, or to revoke their consent and cease participation at any time they wished without giving any reasons. Participants received no monetary, nor any other form of compensation. Their participation in the study was of their free will after receiving extensive explanations on the study. The study was approved by the Ethics Committee of Biomedical Research of the University Mohammed V of Rabat, Morocco, under reference: 96/22.

## 3 Results

Findings showed varied experiences which include (i) biomedical experiences (ii) gendered social experiences (iii) psychological experiences as well as (iv) financial experiences as shown in the following Table 3.

**Table 3:** Themes and subthemes related to the experiences of infertile couples.

Domains	Themes	• Sub-themes
Biomedical experiences of the infertility treatment journey	Lack of clear information regarding infertility care pathway	• Diagnosis not established quickly • Therapeutic management not understood by patients • Information and orientation problem
	Multiplicity of treatments	• Multiple medical treatments • Use of a variety of alternative therapies
	Fractured care or Lack of continuity in care	• Discontinuous monitoring • Frequent change of doctors • Problem of trust in private practices
Social experiences	Gendered social pressures	• Stigma and discrimination • Social isolation • Harassment • Pejorative perception of ART/secretive nature of ART
	Marital and relationship difficulties	• Conflicts and tensions with in laws and marital partners • Threat of potential divorce
	Disruption of daily and professional lives	• Geographic distanceto ART centres • Disrupted labour
Psychological experiences	Psychological suffering	• Negative emotions • Psychological distress
Financial experiences	Financial problems	• High direct medical costs • Indirect costs • Financial indebtedness • Mandatory out of pocket expenditures

### 3.1 Experiences of the treatment journey

#### 3.1.1 Lack of clear information regarding infertility care pathway

Responses from participants indicated that they experienced a lack of clear information on the infertility care pathway. Participants illustrated how diagnosis was not established quickly, with

“It’s a very difficult problem on all levels, a long journey to find out the exact diagnosis, and I was lost in the system.” C1W (38 years old, 10 years infertility)

Participants described the lengthy process that they underwent to identify the cause of their infertility. When asked to describe their treatment journey one participant indicated that it would “*take me days and hours to explain and describe it” C8H (41 years old, 10 years infertility),* while another one indicated that it took her *“5 years to know the exact diagnosis of my infertility” C2W (30 years old, 4 years infertility)*. The search for the reason why there was failure to achieve pregnancy often took years, and consultations with many health providers:

“I consulted many private centers searching for the correct diagnosis. Finally, after a long journey, after several visits to several gynecologists, I understood the cause of my infertility was tubal obstruction.” C4W (26 years old, 5.5 years infertility)

Following the identification of the probable cause of infertility, participants accounts indicated that the precise therapeutic management wasnot understood by patients. Many indicatedthat they did not have an idea of where they were in the overall management plan. For most, it was a “*long journey”,* characterised by *“several consultations” C2H (33 years old, 4 years infertility),* but they didn't understand the overall management plan. In an illustrative example, one male participant when asked to describe what the key treatment milestones were stated that he “*had to go through several stages; the journey is very long, you get lost in the circuit”C1H(40years old, 4 years infertility)*.

It was typical that when asked to describe the stage of treatment they are in, several mentioned that they were lost or confused, as also emphasized by a female participant in another couple:

“It is a difficult experience for most couples, for me the process was very long, and I was lost” C2W

Further examination of participants' experiences suggested that there was an information and orientation problem about infertility and where or how someone can access care, with one participant remarking that:

“The big problem is the lack of information and appropriate guidance. We must communicate about this subject which remains a taboo in society.” C6W (27 years old, 6 years infertility)

Indeed, participants accounts suggested that many couples were not provided relevant information or proper referral process, as illustrated by one participant who when asked how she got to know of the ART center, she responded to state that she had discovered it “*by chance–a woman I met recommended it to me” C3W (30 years old, 9 years infertility).* Although this was not a universal problem, it was common to hear that couples had been searching for information on where to get fertility services. For instance, one participant explained that he *“knew about this center through the internet, when I was looking for more information on this issue…” C13H (44 years old, 13 years infertility).*

#### 3.1.2 Multiplicity of treatments

Multiplicity of treatments was very common among the participants.Firstly, nearly all participants had consulted mutiple health providers or gynecologists. It was common to hear how participants would *“always go to [new] a gynaecologist when someone recommends, or whenever a doctor is recommended” C5W (43 years old, 10 years infertility).* Yet, many had been unsuccessful and had as a result *“spent a lot of time consulting in the private sector, in vain” C4H (44 years old, 5.5 years infertility).* Others had received *“several treatments and several ovulation-induction cycles without success.” C7W (40 years old, 10 years infertility)*.

Secondly, some of the participants had received multiple types of procedures, which included oral treatments as well as surgical procedures, as illustrated by the following quote:

“I had two operations for the removal of an ovarian cyst. Afterwards, I did the laparoscopy. Afterwards, I was treated for tuberculosis. After polyp removal and several interventions and treatments..” C6W

Thirdly, many had sought help from traditional remedies sources outside of healthcare settings, including herbal therapy, blood-letting, witchcraft, and religious blessings. In an illustrative example, one participant reported that she had “*used traditonal methods before:I took herbs as a drink, with no results*.” *C12W (40 years old, 5 years infertility).*

As can be deduced from the foregoing quote, alternative methods were not necessarily successful,yet desperation was a significant factor that caused many participants to resort to traditional methods.

“I did everything, even because of despair…I did “Hijama” [ blood-letting], also Witchcraft “tat9af” “chaaouada”..”[and then]. I changed my approach to move towards the spiritual “Roqia”.” C8W(36 years old, 10 years infertility).

Apart from not being successful some of these methods result in negative health consequences.

An additional reason that contributed to the use of these alternative methods was family members who were said to “*always direct you towards traditional treatment” C12H (43 years old, 5 years infertility)*. Yet, the use of alternative methods was not without risks. For instance, one paticipant who had used herbs, narrated how she *“stopped drinking [herbs] because I had stomach problems…I will never repeat this experience.” C12W.* In other cases, the implications were more serious with some losing consciousness for hours:

“I tried natural recipes..Either recommended by my friends or I searched on the internet. Once I tried a natural recipe and me and my husband lost consciousness from 6:00 p.m. until 6:00 a.m. and from that moment I decided to stop it and follow the doctors’ instructions” C5W

#### 3.1.3 Lack of continuity in care

An important finding wasthat there was a lack of continuity in care, which often led to a longer treatment period. There was discontinuous monitoring, frequent changes of doctors which were often driven by costs of care, or lack of results. Frequent change of doctors, was primarly driven by the search for successful conception. In an illustrative quote one paticipant described how they “*I went to quite a few gynecologists as well as general practitioners…I changed doctors each time to get a result.” C14W (42 years old, 11 years infertility)*. A participant explained how she had “*gone to see more than one doctor in the private sector”,* she further expounded that:

“Whenever I didn't see results, I would go to another one; and when I hear of a doctor capable in situations like mine, I would go immediately to his practice.” C13W (40 years old, 13 years infertility)

The frequent change of doctors was exacerbated by cost of treatment to cause discontinuity of care, as emphasized by one with one participant who stated that *“six years of treatment, six years of medication, is a long time, so we stopped every now and then until we could save some money or borrow it”C11H (39 years old, 6 years infertility).*In many cases, patients drescibed how costs were central to the experience of fractured care:

“We were forced several times to stop follow-up with the treating doctor because of spending all our money and the need to look for other sources to start again: sale of property or loan from a loved one” C10W (25 years old, 5 years infertility)

At the same time, the lack of results, and costs led to psychological impacts and further need to change doctors, leading to a vicious cycle, as illustrated by one husband:

“The psychological state of my wife, added to the lack of results. This pushes me to change doctors each time.” C8H

As a result, some couples had consulted over 10 doctors over several years.

“..And not to mention the number of general practitioners and 13 specialist doctors and famous ones that I consulted with my husband; a long journey of suffering difficult to summarize in a few minutes” C8W.

The frequent change of doctors involved moving across several cities, as illustrated by a couple who had done it to “see a lot of doctors in Beni Mellal, Khouribga, and Oued Zem cities, [but we] still felt frustrated and decided to change doctors again” C10H (39 years old, 5 years infertility).

While frequent change of doctors was common, it emerged that this change was also driven by a lack of trust in private practices by some partcipants, who perceived the private sector pratitioners as being driven by economic gains:

“The interest in the private sector is financial. We didn’t feel supported…..That’s why I changed doctors each time, and when a woman suggests a doctor to me” C10W

Some participants contrasted their current experience in the public ART center with their previous experience in the private sector, noting that *“There are a lot of differences from my experience; the private sector is mainly interested in the financial margin.” C14W*.

**Figure 1:**
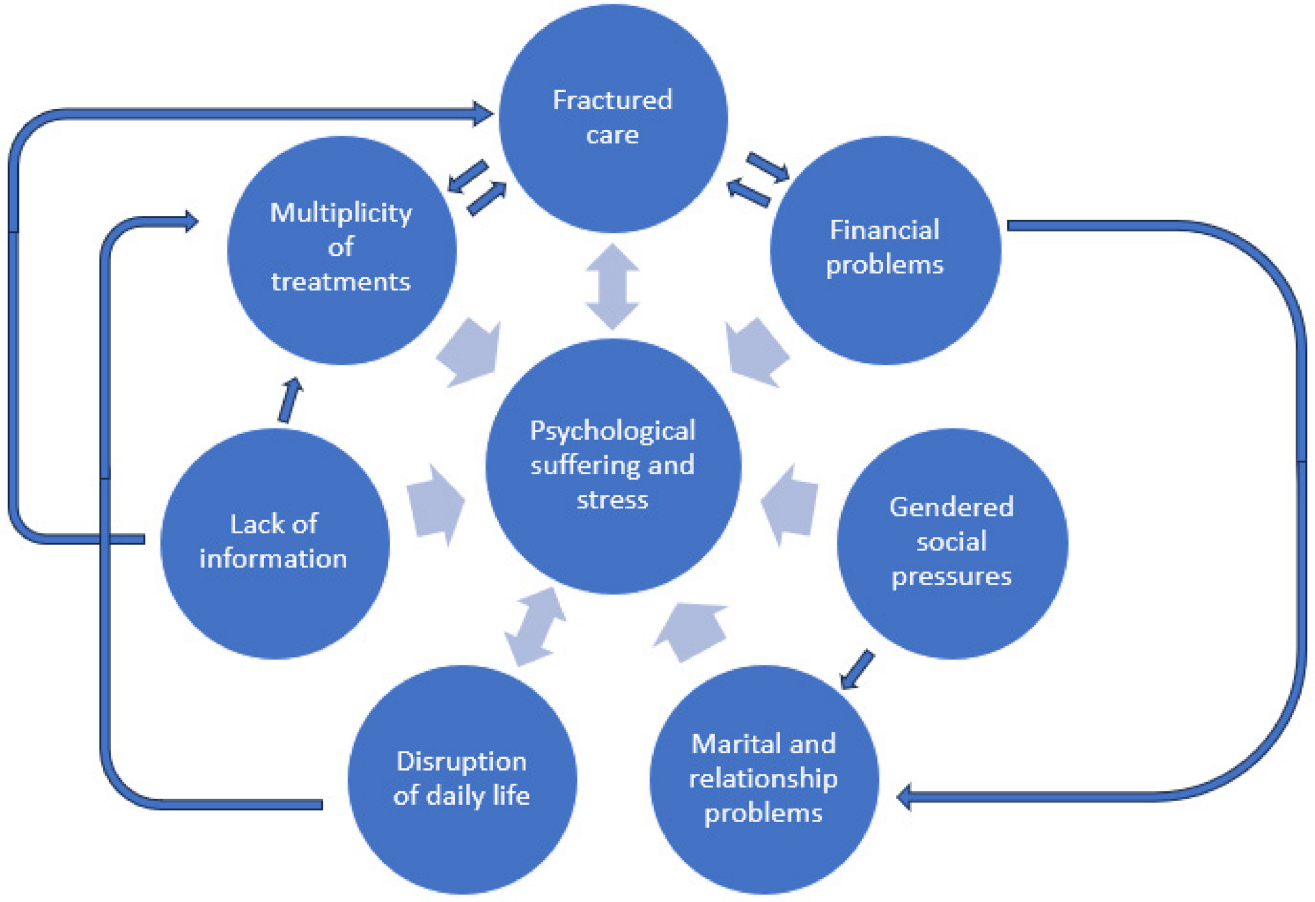
Conceptual framework diagrams showing interconnectedness of drivers of experiences of couples.

Fractured care or lack of continuity in care may often be the consequence of the lack of clear information regarding infertility care pathway and the multiplicity of treatments. Couples undergoing infertility treatments may feel lost, confused, and overwhelmed by the scarcity of information which can lead to frustration and psychological distress and subsequently quitting all treatments (Figure 1).

### 3.2 Social experience

#### 3.2.1 Gendered social pressures

Many participants described experiencing social pressure from families, and in some cases this was described as*“problems with* …* the in-laws”C1W.* The reasons for this were because of the unmet expectations:

“Yes … there is family pressure since we are married and many couples in the family who married after us have had children.” C1H

Stigma and discrimination was a prominent experience among the participants, as illustreted by one participant who described how there is *“a lot of stigmatization in our society regarding this problem” C1W.* Part of this stigmatization was because of *“the general mentality of society, [whereby] children are the pillar of the family in our Moroccan context and any marriage must result in children.” C1H*.

While infertile couples were generally looked down or pitied upon, women were disproportionately affected. A participant noted that *“Being an infertile couple in Morocco means being the victim of all kinds of stigmatization especially the woman” C10W.* Another participant explained that *“Discrimination, stigmatization, society always makes women feel guilty, the feeling of pitying infertile couples” C2W.* Another noted that *“for society the woman is primarily responsible.” C7W.* This participant further indicated that fertility was considered central to marriage in the society's eyes and that an infertile woman was considered *“like an infertile land; without children, the marriage cannot continue…C7W*. In this context, *“the society considers you disabled, incomplete “nakssa”..” C10W.* Another participant noted that *“sincerely, the experience of an infertile woman is hard, we call the woman “Aagra” [incapable of giving birth]… It touches me deeply, but I try to resist people's words..” C6W.*

As might be noted from the above quote, many stigmatized individuals tried to ignore pejorative labels, as a way of coping with their situation, while chose to isolate themselves as illustrated by the following quote:

“Because of a lot of stigma, discrimination, I stay alone to avoid questions from relatives on the subject.” C3W

Asked to explain further, this participant explained as follows:

“I avoid visiting family and especially during happy events weddings or “Sboua” baptisms. I prefer to stay alone to avoid questions from family or acquaintances on the subject.” C3W

Indeed, many participants, particularly women described the harassment in forms of inquisitive questions that made it difficult for them to take part in social events with families or friends:

“A lot of people ask you questions all the time: “why haven’t you had children until now?”, some recommend going to this or that gynaecologist, I feel all the time that they have much pity of me.” C4W

While some of these questions were accompanied with suggestions of whether help could be gotten from, others were perceived as being accusing women of not wanting to have children as re-counted by one participant:

“Sometimes I receive questions like “you haven’t gotten pregnant yet? Aren’t you thinking about having a baby?”. I feel very bad because they think I can, but I'm the one Who doesn't want a baby. I have become very sensitive about the subject, and I always try to change the subject.” C5W

Rather than just describing isolation as a means of coping, several described experiencing social isolation as the result of these enquiries, which were described as traumatizing:

“It is a source of social isolation, from being traumatized at each meeting with family or friends by the question about pregnancy and the reasons for the delays and the forced proposals for remedies, treatments, doctors, recipes, without any respect for my private life or my intimacy. I decided to no longer participate and respond to invitations…I prefer to stay in my quiet corner, society is difficult and people are too aggressive” C10W

An interesting finding related to these inquisitions and the stigmatized nature of infertility was that treatment was kept secret and often not disclosed to family. Participants seemed to suggest that there was apejorative perception of ART treatments, mainly because infertility *“still remains taboo for uneducated people and for the majority, stigma..” C2H.* A participant noted that *“the majority of society is ignorant of the existence of these sophisticated and modern ways of helping couples have children” C11H (39 years old,6 years infertility)*. For this reason, many in the society cast doubts about whether those attending treatments would be sure that the child they got through IVF was really their biological child. This male participant further explained that:

“I have decided that we will not tell anyone about this process to avoid any embarrassment and the overwhelming question: ‘how do you know it’s your baby?’” C11H

Indeed, it was common to hear that couples that were attending treatment had *“decided not to tell the family”* C12H, which added on their experience of secrecy around infertility treatment, particularly IVF. Others did not want to deal with the perception of failed IVF, and noted that they *“didn't tell anyone that I would do IVF at first, because I wanted to see how it would go first” C5W.*

#### 3.2.2 Marital and relationship difficulties

Apart from secrecy within families, infertility also had an impact on couples themselves. Accounts from several participants, mostly women indicated that their relationships with their husbands had deteriorated. One stated that she “*had a lot of problems with ‘[her] husband..” C1W.* In some cases, these problems occurred when there was some friction with the women's in-laws:

“A lot of problems arose with my husband, a little from the in-laws..” C4W

These narratives depicted images of women that were being blamed, and in some cases, about to be deserted by their husbands.

“[We had] problems as a couple,and we agreed to go for divorce in the event of IVF failure..” C8

Having mentioned that, not all participants indicated a deterioration of relations, and at least two participants indicated that their husband had been supportive. One woman described how her *“husband and his family have been very understanding” C5W*, while another noted how this was important in a context where men faced pressure from their families to marry someone else:

“Luckily my husband’s family supports me, and they don't ask him to marry another woman….Because in Moroccan society people get involved in these things even if they are personal and sensitive. They always show a woman who is late to become pregnant as imperfect, incomplete without children…and she is the guilty one.”C14W

Overall, it was clear that that gendered social pressure was more focused on women and therefore seemed to have greater impact on women's emotional and psychological state compared to men, while at the same time had an impact on marital relationships (Figure 1).

#### 3.2.3 Disruption of daily and professional lives

Given the frequent travel to treatment centers, an important finding was the disruption of daily and professional lives that the frequent appointments required. Participants frequently note that although cost was an issue, *“distance also poses a problem” C6W.* This was particularly important considering that the public ART center in Rabat serves as a national referral center, serving patients from across the country. Indeed, participants described that many “*…come from cities that are very far away; we too travel from Casablanca to Rabat..” C13H. Others had to travel for hours and had to “take the early 6am train so I can be on time..” C13W.*

In addition, findings suggest that the multiplication of travels caused disrupted labour affecting the professional and the daily lives of couples seeking infertility treatments, as was reported by a female participant:

“I was a primary school teacher far from my town in the mountains….and I always travelled when someone recommends a gynaecologist or I hear a doctor is recommended…As I had a lot of work, I gave up…” C5W

### 3.3 Psychological experience

#### 3.3.1 Psychological suffering

Psychological suffering was another central hallmark of participants' experiences. Participants indicated that they “*suffered a lot psychologically, of all kinds, stress, and anger”C1W,* while others emphasized that infertility *“causes lot of stress, isolation, tension, and bad mood”C3W.* Others described how they either *“experienced depression, difficult times and suffering.” C7W, “suffered from depression” C10W or became “completely hopeless and depressed” C8W*. Anxiety, stress and anger were very common cross all the participants, and were often mentioned together by participants, as illustrated in the following quote:

 “Every time I think about it, I feel a lot of anxiety, stress and anger.” C2W

These negative psychological experiences were aggravated by the fact that time was passing which was perceived as reducing the possibilities for successful conception.

“It’s very difficult, very stressful, especially time passes quickly and with age the chances of pregnancy decrease. A lot of stress, anxiety” C4W

Psychological suffering was also exacerbated by failure of treatment, and participants mentioned repeatedly that they “*felt a lot of suffering and stress in the face of the lack of good results..” C4H.* Others mentioned how they felt *“anxious, sad and irritated, disappointment after several treatments without results.” C6W.*

In general, psychological suffering was not perceived as part of men's experience; it was mainly related to the woman in the couple. Indeed, some men themselves alluded to this, as illustrated by one male participant who asserted that as follows:

“Personally, I don't see late pregnancy as a problem in my life and it doesn't hurt me as much as my wife..” C11H

Although psychological suffering was mainly noted among the women, it seemed that beneath the surface, men also experienced it, despite it not perceived as such. One participant explained that men also “*men also suffer but in silence!” C12H*.

### 3.4 Financial experiences

#### 3.4.1 Financial problems, indebtedness, and out of pocket expenditures

Financial pressure was a common and difficult experience that early all participants underwent. In general, this was because the treatment is “*very expensive” C1H.* Participants stressed that treatment *“is expensive [especially] in the private sector”C3H*. Not surprisingly, cost was perceived as a significant barrier preventing participants' access to healthcare as illustrated by the following quote:

“The financial aspect is the first obstacle for treatment” C12W

Finances caused stressful experiences among participants. Even at the public ART center, participants were still expected to partially contribute to treatment costs, yet many did not have medical insurance:

“It’s very difficult, especially when there is no medical coverage..the cost of medication is very expensive and so are the tests.” C3W

Subsequently, out of pocket expenditures were common among all the participants, who noted that treatment “is very expensive in the absence of medical coverage” and that “since we have no health coverage or medical insurance, we pay for everything without any reimbursement” C13. Indeed, many of these couples described how they had borrowed money from banks, family, and friends. For example, one described how they “spent a lot of money, sometimes from loans, savings..or selling their goods to pay for [treatment] services..” C8. These findings suggested that financial protection could mitigate financial pressures, as also emphasized by participants:

“Since the majority of people do not have the capacity to pay treatment costs, it is absolutely necessary that infertility be included as an illness covered by social security. It’s suffering in silence!” C11H

Cost of treatment was exacerbated by indirect costs, including travel and accomodation. Several participants stated “*adding the mobility costs to the financial aspect of the treatment makes it more complicated” C13H.* A key issue in these indirect costs was the distance between the ART center and where the participants lived, which increased the financial requirements:

“..Financially it is too hard, especially for us from distant regions…..The problem is the distance: My husband and I had to travel from far away, we had no accommodation, nor the means to have one..” C7W

As noted above the financial problems was also connected with the lack of decentralised services, many travelled from far, yet this was also connected with disruption that couples experienced as they travelled for care. Not surprisingly, participants suggested that decentralised treatment can ameliorate this problem.

“There are also transport and travel cost: if a center like this exists in my city, we will save on mobility costs.” C13W

### 3.5 Potential solutions to improve experience

Given the participants descriptions of their experiences, some described as possible solutions to improve their experiences, including, enhancing public awareness and education around infertility, creating, and linking people with infertility to support groups of other people with infertility, enhancing financial protection, for example through insurance schemes or free provision of services, and decentralising services by creating other similar centres across the country. It was noted that couples were having empathetic care which they emphasised should be continued (Table 4).

**Table 4:** Possible solutions to improve couples experiences.

Domain of experience	Potential solutions	Illustrative quote
*Biomedical experience*	Continued empathetic care	• “The center team surprised us with their professionalism, presence and empathy” C10W • “The explanations in a very simple and appropriate way. Listening and answering our questions.” C10W • “Members of the team are very empathetic and communicate very well” C6W
*Social experience*	Enhance public awareness and education on infertility	• “The first thing is awareness in the media for all couples in Morocco..” C1H
	Create support groups for people with infertility	• “..I tried to have contact with another woman in the same situation to support each other” C8W
*Psychological experience*	Include psychosocial element in services	• “Psychological support is essential for couples; I would say it's everything!” C12W • “We must provide guest houses near the center to improve geographical accessibility to the center, also hotels at symbolic cost, as well as multiplying public ART centers across the Kingdom.” C1H
*Financial experience*	Provide financial protection	• “Establish a free system and that the state gives priority to couples suffering from infertility” C1H • “Establish and generalize medical coverage in the field of ART especially for couples with limited resources” C4H
	Decentralise services to other parts of the country	• “Multiply similar centers and improve geographic accessibility for other regions and several cities to limit the travel of people residing in distant cities” C3H • “The government can help us with health coverage and create many centers in many cities like the rest of the countries.” C11H

## 4 Discussion

This paper reports the experiences of couples with infertility in Morocco, which is rarely researched in the country. Results provide rich insights showing a wide range of experiences among the participating couples. Several hallmark features of couples' experiences were identified that warrant further discussion.

First, there was an information and orientation problem, which was associated with a lack of a clear infertility care pathway, diagnostic delay, and lack of information regarding treatment plan. Patients were not involved nor informed what their treatment journey would entail. Research from other countries indicates that there is a lack of adequate information and preparation of patients so that they would know what the diagnosis and treatment entails. This is a significant part of patients' experiences of infertility (25), and while numerous studies indicate that patients desire to be informed about their planned treatment (26), health providers do not always involve them in such discussions (27).

These findings suggest that patients should be counselled, informed, and discussed with, regarding their treatment journey. It is important to clearly inform patients what the therapeutic management is so that they can know what the first option, second option or other advanced options are. Although Morocco has national guidelines (a national guide for the management of infertile couple was developed in 2016), it is unclear if these are followed by all stakeholders, including all gynecologists in both the public and private sectors. It would be important to sensitize health professionals about the national guidelines and insist that they communicate with patients about what the diagnostic and treatment strategy would be expected to be, based on these guidelines. Fundamentally, it is important to raise awareness among health professionals about the importance of information and communication with infertile couples.

Second, our study shows that there was lack of continuity of care and multiplicity of treatments. Repetition of treatments and procedures has been reported in other settings, but in our study setting, this was particularly driven by frequent change of doctors among participating patients who were frustrated or seeking second opinion, as well as drop off from treatment due to financial problems. While many of the participants interviewed in our study were accessing fertility care, unfortunately, in many low- and middle-income countries as shown in studies conducted in the Gambia (28), Kenya (29), South Africa (30),, Zambia (31) and other countries access to infertility services, including specialized interventions such as IVF, is very limited, and it can be anticipated that access to care could also be fragmented in those countries. This is particularly relevant given that IVF is expensive and unaffordable in many low income settings (5), which also prevents its inclusion in public policies and services (4, 32).Yet financial costs have been shown to contribute to stressful infertility experiences (33). These findings suggest that enhancing financial protection through inclusion of infertility treatment in health insurance schemes, or reimbursements of costs would improve patients' experiences and enhance continuity of treatment.

Third, the use of traditional therapies such as herbal therapy, bloodletting, witchcraft, and religious blessings was identified as a contributor to some negative experiences, for example gastrointestinal side effects. Given that some of these were driven by desperation, there is a need for tactful messages to couples to lessen psychological impact in case of poor prognosis, while at the same time educating patients about the potential risks of use of some traditional therapies. Use of traditional or folk therapies has also been reported in other low income (34, 35) rarely, in high income (36) settings, often because they are cheaper compared to modern medicine. In our setting, it is not clear whether the risks of some of the folk therapies were known to participants. This points to the need for IEC (Information—Education—Communication), for example organized information campaigns in basic health care facility in order to enhance awareness among couples on the potential danger of traditional therapies. Health professionals should devote some time during their daily practice to counseling about these practices.

A surprising finding was that patients had mixed experiences with the private sector, which was partly related to financial implications of treatment. While nearly all the participants had attended a private practice previously, there was tension in that private sector, doctors were sought for their expertise, yet there was a problem of trust with private practices in general. There was a perception that private doctors may recommend expensive treatments when not needed. This is consistent with reports from other countries where private sector is perceived as driven by economic motivations (28, 37, 38). Yet, the private sector is an important part of the health system in Morocco, and it is important that all practitioners follow the guidelines, standard operating procedures.

Fourth, negative psychosocial experiences, which are often gendered were common part of the couple in this study, including stigma and discrimination, isolation, as well as marital and relationship difficulties, which disproportionately affected women. Numerous other studies have shown the gendered way in which infertility is experienced, which is consistent to our findings (39). Besides this gendered experience, overall, a good number of participants in our study reported a variety of emotional and psychological distress experiences, including depression, anxiety, anger. Similar findings have been demonstrated in other countries showing widespread experiences of negative social pressures (8), and relationship problems (40) as well as emotional psychological distress among patients with infertility (40–42), which are often linked to widespread stigmatization of infertility in communities, for example in Malawi (43), Zambia (31), Bangladesh (44) and numerous other countries. In our study, and in attempts to cope with the societal pressures and stigma, participants sought to keep their treatment a secret, which was itself an additional psychological burden, a finding that supports previous assertions that treatment itself can be an important source of distressing psychological experience (6, 40).

This finding suggests that there is the need to destigmatize infertility in Moroccan communities. There is a role for using media channels (TV, Radio, social media) to enhance the understanding of infertility and destigmatize it. IEC is needed to ensure that communities understand that infertility is a preventable and treatable condition. A lack of information on infertility is rampant in many counties, and researchers have identified the need for awareness raising campaigns to destigmatize infertility (45) and assist in help seeking (46). Apart from destigmatizing it, efforts are needed to strengthen psychological support as part of the management of infertile couples. Social and psychological support is not included in the national guidelines, and therefore needs to be strengthened in the future. An important practical step would be to ensure that the national guidelines are expended to include a psychosocial section.

In addition, to operationalizing psychosocial support, it would be important to ensure that a psychologist is included as part of the team of every fertility center. Where this is not feasible, focal health care providers should be trained on psychological counselling. Studies from other countries have shown that this is an important part of management that could reduce psychological, relational and social impact of infertility (40), including gendered-stigma (39). Another practical step that can be implemented is the creation of patient support groups which can provide members with a sense of empowerment, help sharing coping strategies, provide a sense of community. This is particularly important given that many participants felt lonely and isolated, and relied on social support from families, spouses, or friends, which was not always structured or forthcoming. Studies suggest that it is often difficult to create support groups for stigmatized conditions, but where these exist, they enhance the capacity of members to resist or cope with their diagnosis (40). Based on the findings of a recent review of effective psychosocial interventions (39), these kinds of interventions will require to be tailored to cultural norms of the targeted settings, for example ensuring that the gendered experiences target reshaping of masculinities.

In addition, our findings suggest the centralized nature of IVF treatment creates both financial and disruption of social lives of participants. A potential solution to this issue is the creation of centers in several regions of the country. At present there are only 3 Public ART centers, which serve the entire country as referral centers. A practical way of decentralizing these services is the creation of an ART center in every University Hospital meaning one for each region. This will ensure the training of health professionals on ART in decentralized centers and that patients will not have to travel as far as they do now. Indeed, as shown in Table 5, a range of implications for policy and practice could be considered.

**Table 5:** Issues faced by infertile couples and implications for policy and practice.

Issues faced by infertile couples		Implications	
	Objective	Policy	Practice
Lack of clear information regarding infertility care pathway, multiplicity of treatments and fractured care	Improve patients' experiences and quality of infertility care services.	1. Establish an updated evidence-based national or territorial health groups' guidelines for infertility management Including • Diagnostic tests, • Therapeutic options psychosocial care, • Follow-up services 2. Develop primary, secondary, and tertiary care coordination ensuring a continuous step-by-step management of patients	1. Healthcare providers should draw up a holistic care plan including all aspects of infertility (medical, psychological and financial) 2. Healthcare providers should benefit from a training on standardized clinical practices, including how to communicate and share information with patients concerning treatment options, cost, duration, and possible outcomes. Provide training on how to manage the emotional burden borne by patients.
Gendered social pressure	Reduce infertility stigma	1. Improve public perception by education, information, and communication campaigns 2. Ensure that availability of services for both female and male causes of infertility	Integrate couple-based counselling, group therapy and support programmes promote public awareness.
Psychological suffering	Reduce psychological stress and burden faced by people with infertility	Enhance inclusion of psychosocial and mental health elements in fertility care	Implement in all infertility care services with mental health care aspects including: • Stress management • Couple counselling and conflict management • Peer support groups • Group therapy
Disruption of daily and professional lives	Decrease patients' struggle and travel time to access treatments	1. Creating other more ART centres across the country 2. Paid leave for infertility treatments	Provide basic services in each Centre and refer patients for complex cases
Financial problems	Enhance financial accessibility	1. Extend insurance coverage to infertility treatments 2. Develop Standardized treatment costs	Inform couples in advance about their financial options to be able to make decisions

Finally, many of the experiences of our participants are interconnected, and these interconnections spanned by individual, interpersonal, social and structural domains. One the one hand, the social and gendered distinction between both partners caused emotional distress among women, could trigger marital and relationship difficulties, and exacerbate psychological suffering. On the other hand, psychological suffering often occurred due to social isolation, but was also linked to lack of sufficient information about treatments, the multiplicity of treatments or financial problems. Financial pressures often led to multiplicity of treatments, which was linked to linked to the disruption of care and the disturbance of professional lives, particularly because of the distances involved in accessing care (Figure 1). The financial problems and disrupted care affected couples emotional well-being leading to a feeling of helplessness and other psychological problems. The existence of gendered social pressure impacted the marital experiences of couples undergoing infertility treatments by amplifying their distress, both of which contributed to psychological suffering. These experiences overlap in their influences, and often intensify the overall experience of couples seeking infertility treatments as shown in Figure 1. Our finding of interconnectedness of these influences is consistent with a recent study demonstrating intersecting drivers of experiences of infertility among women in India (47), and implies the need for a holistic approach when addressing all the issues faced by the couples during their treatment journey (Table 5).

## 5 Limitations

This study is not without limitations, the main one is limited generalizability. Given that the study included a relatively small size sample recruited from a single Public ART Center and in the capital city, the results might not reflect on the experiences and perspectives of a larger population of infertile couples, it also might not show regional and cultural differences. However, the Center serves as a referral center for the country which could mitigate this limitation. Additionally, our primary intention was to show diverse and representational examples of experiences, through qualitative enquiry. Nevertheless, given that ours constituted a convenient sample, recruited consecutively based on their routine clinic appointments, it is possible that the research teams positionality, and the situatedness of the study could have influenced what participants diverged (23).

Although extensive thematic analysis was conducted through the aid of software, it is possible that some relevant themes may remain unidentified (23).Moreover, all the participants had sought treatment at the center which excludes all the infertile couples who did not want or could not seek treatment; therefore, our study excludes the possibility of themes, alternative experiences and complex or diverse viewpoints from those not accessing treatments. Nevertheless, our study provides useful learnings that may inform improvement of experiences of couples with infertility and may represent a basis for future research. In future, consideration of multicentre studies involving ART centres from various regions in Morocco, along with their varying health system and cultural contexts could provide additional learnings relate to experiences of infertility, by capturing a broader range of experiences and perspectives. Additional study designs that can be considered may include use of a longitudinal design which can provide information on experiences over time. Although our studies used interviews, the combination of interviews**,** focus groups**,** and observations may add more insight where these methods complement each other.

## 6 Conclusion

This paper reports the experiences of couples with infertility in Morocco, which is rarely researched in the country and other low- and middle-income settings. Results provide rich insights showing the wide range of experiences that couples with infertility go through, which include biomedical experiences (diagnostic delays, fractured care, multiplicity of treatments), gendered social experiences (gendered social pressures, stigma, discrimination, isolation and disrupted social routines) psychological experiences (anger, anxiety, depression, and mood changes) as well as financial experiences (out of pocket payments and financial indebtedness). The results of this research indicate the urgent need for a wide range of health systems strengthening strategies to improve the experiences of people with infertility, including the financial protection, service decentralization, public education campaigns to educate the public and destigmatize infertility, social support groups for people with infertility, and enhanced psychological counselling as part of routine fertility care provided to infertile couples. Given interconnectedness between biomedical, social, psychological, and financial divers of experiences, a holistic approach is required to comprehensively improve the situation of infertile couples.

## Data Availability

The raw data supporting the conclusions of this article will be made available by the authors, without undue reservation.
