# Supplementary material for: Experiences of infertility among couples in Morocco
Source: Front Reprod Health. 2025 Jan 7;6:1513243. doi: 10.3389/frph.2024.1513243 (PMC11753203; doi:10.3389/frph.2024.1513243)
Supplement: Supplementary file 1 [file Datasheet1.pdf]

Interview Guide for Men and Women with Infertility

Participant Code NUMBER: ________________

Date of Interview: ____ / ____ / ____

Site of Interview: ______________

Result Code:

1. _____Finished
2. _____Refused
3. _____Partly Finished

Interviewer Code: ________

Interviewer Signature: __________________

1. Demographic profile

To start with, I will ask you a few questions about yourself:

What is your age?	
What is your gender	Male ☐<br>Female ☐
What is your place of residence?	
What is your marital status?	
What is your highest level of education?	Illiterate ☐<br>Primary ☐<br>Secondary ☐<br>University ☐
What is your profession?	
How long have you been trying to achieve a pregnancy with your spouse?	
How many children do you intend to have in life?	
Do you have health insurance	Yes ☐<br>No ☐
If you have insurance, which one?	RAMED ☐<br>CNOPS ☐

	CNSS ☐<br>Other ☐
.	

2. **Experience with infertility prior to coming to this ART Center**

   Now, I would like to ask you a few questions about your experience with infertility before you came to this center.

   2.1. What is it like to have infertility in Morocco?*[Researcher: Probe Context]*

   2.2. How did you experience your infertility before your consultation in this center?

   2.3. At psychological level?*[researcher to probe stigma, mental health, anxiety, mood]*

   2.4. At economic level?*[researcher to probe effect on finances, household savings, loans]*

   2.5. At the family level?*[researcher to probe effect on relations with spouse, in-laws]*

   2.6. At the Social level?*[researcher to probe stigma, discrimination, exclusion, etc]*

3. **Help seeking and first impressions**

   3.1. How did you come into contact with this ART Center? *[researcher to probe: How did the participant obtain information about this Center? Did they consult any friends or relatives or professionals and asked for their recommendations?]*

   3.2. What were your impressions and feelings the first time you learned about the possibility to visit this ART center?

   3.3. What were your expectations before starting your care at this center?

4. **Experiences of accessing care at the ART Center**

   4.1. What was your experience during your treatment at the center? Were your expectations met? How so?

   4.2. What is your opinion about the care that you are receiving at the Center?

   4.3. Are you satisfied with the quality of your care at this public center:

   - Information yes ☐ No ☐
   - Communication yes ☐ No ☐
   - Health professionnal's support yes ☐ No ☐
   - Medical care yes ☐ No ☐
   - Financial accessibility yes ☐ No ☐

   4.4. Was the nursing consultation beneficial for you?

   Yes No

   4.5. Why?

   4.6. Have you at any point in time considered stopping treatment from this center? Why?

4.7. How much money have you already spent on diagnosis and treatment? Where did you obtain those funds from? What helped you to cope with the financial pressures?

5. **Benefits of a public ART Center**

5.1. Had you attended a private clinic prior to coming to this ART center?

Yes

No

5.2. If so, were there any differences you noticed between the public ART Center and the private ART Centers? If yes, what were they?

5.3. In your opinion, do you think that the ART centre is having an effect? Which one?

5.4. Would you recommend the Center to your family and acquaintances? why?

5.5. What kind of people do you think would benefit most from a public ART Center and why?

5.6. In your view, which factors are contributing to the Center having an impact? How do these factors cause the Centre to have an effect? In what way? [Probe Mechanisms]

5.7. What do you think are the reasons why people could be coming or failing to come to this ART Center?

5.8. How can this center improve its services to other people in Morocco?

5.9. Do you think that people in other countries should have a Centre such as this and why?

Thank you very much, that is the end of the interview. I will stop the recording now.
