# Supplementary material for: Experiences of infertility among couples in Morocco
Source: Front Reprod Health. 2025 Jan 7;6:1513243. doi: 10.3389/frph.2024.1513243 (PMC11753203; doi:10.3389/frph.2024.1513243)
Supplement: Supplementary file 2 [file Datasheet2.pdf]

Interview Guide for Policy Makers

Participant Code NUMBER: ________________

Date of Interview: ____ / ____ / ____

Site of Interview: ______________

Result Code:
1. _____Finished
2. _____Refused
3. _____Partly Finished

Interviewer Code: ________

Interviewer Signature: __________________

1. General Landscape and Context of Fertility in Morocco

First, I would like to start by asking you a few questions on the general situation in Morocco around infertility.

1.1. How is Morocco as a country addressing infertility?
1.2. Do we have a national policy or a strategic plan to address infertility in Morocco?
1.3. Which policies and laws regulate fertility care and assisted reproduction in Morocco?
1.4. Is there a national registry and licensing body for fertility care and assisted reproduction?
1.5. Is infertility included as an essential component of Sexual and Reproductive Health and Rights (SRHR) policy and services in Morocco?
1.6. Do you think that fertility care is important in our setting and why?

2. Setting up of the Public ART Center

I would now like to talk with you about the first Public ARTC Center at XX which you may have been involved in setting up or have at least heard about.

2.1. What was your role in the implementation of this first public ART center in Morocco?
2.2. What was the situation like before the first public ART Center was put in place? How were couples accessing services? What problem did it solve?
2.3. What steps were taken to ensure that services could be started at the ART Center? [please elaborate]
2.4. What policies and regulations were needed to ensure that ART provision was possible at the Center? [please elaborate]
2.5. What are some of the actions and measures that were needed in order to enable the provision of fertility services in Public ART Centers? *[Researcher to probe what action*

needed to take place in relation to i) Pricing of ART treatments, ii) health coverage of infertility treatments, iii) Marketing Authorization and Registration of culture media and medical devices, iv) standardization of public ART centers, v) development of infertility management guideline, vi) Integration of infertility in Health Plan, and v) Application decrees of Bill N° 47-14 on ART]

2.6. What were the key investments in the health system infrastructure that needed to be made during the setting up and implementation of the Center? *[Researcher to probe what action needed to take place in relation to i)service delivery, ii) health workers iii) health records iv) infertility medicines and equipment) management and leadership and vi) financing and subsidization]*

2.7. How is the setting up of the provision of public fertility care at the Public ART Center financed? Please elaborate.

3. Contributions and Outcomes of the Public ART Center

I would like now to focus on events since first Public ART Center was set up.

3.1. What difference do you think the ART Center has made to people with infertility? Why so?

3.2. Who do you think is benefiting from the Center? *[Researcher probe if the Center is benefiting people from all regions, social economic status, ethnic or religion etc.]*

3.3. Why do you think the ART Center is mostly benefiting these people?

3.4. In your view, which factors are contributing to the Center having an impact? How do these factors cause the Centre to have an effect? In what way? *[Probe Mechanisms]*

3.5. In your view, what factors can potentially prevent provision of fertility care services for men and women with infertility at the Center? What should be done about these issues?

3.6. Compared to existing need, do you think that the Center is meeting the needs of fertility care in Morocco? What else should be done?*[Researcher to probe further, researcher might point out that the Center is in a large city. How does this affect rural population? Are the number of ART Centers adequate?]*

3.7. In your opinion, does the ART Center play any other role in fertility care provision? Which one? [researcher probe referrals or training of health professionals]

3.8. What are the reasons why the development and implementation of the ART Center has been successful or not?

4. Perspectives on learning from Morocco to other countries

I would like to finish off by asking you about what has been learnt in Morocco and how it can be used to assist other counties to start provision of fertility care in public hospitals.

4.1. In your opinion what would be the benefits, if any, to the implementation of a publicly funded ART Center in another country?

4.2. In your opinion, are there obstacles to the development of public ART Center? If so, which ones and how can these be overcome?

4.3. What other considerations do you think should be taken into account if/when introducing such public ART Centers in other low- and middle-income countries?

Thank you very much, that is the end of the interview. I will stop the recording now.
