# Supplementary material for: Experiences of infertility among couples in Morocco
Source: Front Reprod Health. 2025 Jan 7;6:1513243. doi: 10.3389/frph.2024.1513243 (PMC11753203; doi:10.3389/frph.2024.1513243)
Supplement: Supplementary file 3 [file Datasheet3.pdf]

Interview Guide for HealthCare Providers

Participant Code NUMBER: _________________

Date of Interview: ____ / ____ / ____

Site of Interview: _______________

Result Code:

1. _____Finished
2. _____Refused
3. _____Partly Finished

Interviewer Code: ________

Interviewer Signature: ___________________

I would now like to start recording our conversation.

1. What is your profile?

   MD

   Nurse

   Lab technician

   Other (if other please specify)

2. How many years of experience in this role do you have?

   <5 years

   5 to 10 years

   > 10 years

3. Please tell me about various fertility care services (e.g., preventive, diagnostic, and treatment) available at your clinic/department *[researcher to facilitate the conversation according to the services.]*

4. Why are these services important in our setting?

5. What is your role in the management of infertile couples within the ART unit?

6. Is there a basic training in the management of infertile couples and ART in Morocco?

7. Have you received a training in the management of infertile couples and ART?

Yes

No

8. If yes, could you tell me where, for how long, what were the objectives of the training?

9. What was the situation like before the ART Center was put in place? How were couples accessing services? What problem did the ART Center solve?

10. What were your contributions in the implementation of the first public ART Center?

11. During the implementation of the ART Center, did you face any challenges?
    Yes

    No

12. If yes, what were they and how did you overcome them?

13. What were the achievements of the implementation of the ART Center?

14. Since the creation of the ART Center, did you contribute in any way in the improvement of the management of infertile couples in Morocco?
    Yes

    No

15. If yes, How?

16. Do you think that the Centre is having an effect? Which one?

17. Which people do you think is being affected most (positively or negatively) by the Centre? Why is that? *[Probe Context and Mechanisms]*

18. In your view, which factors are contributing to the Center having an impact? How do these factors cause the Centre to have an effect? In what way? *[Probe Mechanisms]*

19. Compared to the need, what is your view on the availability of Government hospitals and public ART Centers that can treat couples with infertility? *[To probe further, researcher will point out that most of these services are in large cities and need more services.]*

20. What else needs to be done to increase couples' access to preventive, diagnostic and therapeutic interventions for infertility?

21. What are the three most important lessons you learned from your experience in the implementation and thereafter the clinical management of the ART Center?

22. According to your experience, what would be your recommendations to other low- and middle-income countries if they want to implement public ART Centers?

Thank you very much, that is the end of the interview. I will stop the recording now.
